# XB130 is overexpressed in prostate cancer and involved in cell growth and invasion

**DOI:** 10.18632/oncotarget.11074

**Published:** 2016-08-05

**Authors:** Bin Chen, Mengying Liao, Qiang Wei, Feiye Liu, Qinsong Zeng, Wei Wang, Jun Liu, Jianing Hou, Xinpei Yu, Jian Liu

**Affiliations:** ^1^ Department of Science and Training, General Hospital of Guangzhou Military Command of People's Liberation Army, Guangzhou, Guangdong, China; ^2^ Guangzhou Huabo Biopharmaceutical Research Insititute, Guangzhou, Guangdong, China; ^3^ Department Of Pathology, Peking University Shenzhen Hospital, Shenzhen, China; ^4^ Department of Urology, Nanfang Hospital, Southern Medical University, Guangzhou, Guangdong, China; ^5^ Cancer Center, Traditional Chinese Medicine-Integrated Hospital of Southern Medical University, Guangzhou, Guangdong, China; ^6^ Department of Urology, General Hospital of Guangzhou Military Command of People's Liberation Army, Guangzhou, Guangdong, China; ^7^ Sun Yat-Sen University, Guangzhou, China; ^8^ Guangdong Provincial Key Laboratory of Geriatric Infection and Organ Function Support and Guangzhou Key Laboratory of Geriatric Infection and Organ Function Support, Guangzhou, Guangdong, China; ^9^ Center for Geriatrics, General Hospital of Guangzhou Military Command of People's Liberation Army, Guangzhou, Guangdong, China

**Keywords:** XB130, adaptor protein, proliferation, invasion, Akt

## Abstract

XB130 is a cytosolic adaptor protein involved in various physiological processes and oncogenesis of certain malignancies, but its role in the development of prostate cancer remains unclear. In current study, we examined XB130 expression in prostate cancer tissues and found that XB130 expression was remarkably increased in prostate cancer tissues and significantly correlated with increased prostate specific antigen (PSA), free PSA (f-PSA), prostatic acid phosphatase (PAP) and T classification. Patients with highly expressed XB130 had significantly decreased survival, which suggested XB130 as a possible prognostic indicator for prostate cancer. *In vitro* experiments showed that reduced XB130 expression restrained tumor growth both *in vitro* and *in vivo*. Furthermore, XB130 knockdown hindered transition of G1 to S phase in prostate cancer cell line DU145 and LNCap, which might contribute to the inhibition of cellular proliferation. Results from transwell assay demonstrated that downregulation of XB130 may attenuate invasion and metastasis of prostate cancer. Semiquantitative analysis of Western blot suggested that decreased XB130 expression was accompanied by diminished Akt signaling and EMT process. Thus, above observations suggest that XB130 may be a novel molecular marker and potent therapeutic target for prostate cancer.

## INTRODUCTION

Prostate cancer is the most common and second lethal cancer in western male population [1, 2]. PSA is the major significant marker for the diagnosis of prostate cancer with a low specificity of 18.67%–30.08% as single use in for biopsy [3]. To clarify the molecular mechanism involved in prostate cancer is of great importance for finding a potent and more effective diagnostic marker and improving the treatment of prostate cancer.

Adaptor protein has a unique modular structure without enzymatic activity, which can be regulated by multiple signaling. It consists of actin filament associated protein (AFAP), Src interacting/Signal integrating protein (Sin) and Crk-associated substrate (CAS). Multiple studies have proved this functions in mitosis, differentiation, inflammation, cell survival, movement, and adhesion through binding SH3 and SH2 domains and activating c-Src [4–6]. AFAP, as a small adaptor protein, is involved in cell signal transduction, assembling of cell cytoskeleton and other cell functions, which can directly induce activation of c-Src by mechanical stretch. XB130, also named AFAP1L-2, is a 130kDa adaptor protein with 818 amino acids located on chromosome 10q25.3 [7]. As the substrate and regulator of signal transduction mediated by tyrosine kinases, XB130 phosphorylates tyrosine through SH2 and SH3 domains at the N-terminal of Src [8]. The role of XB130 varies in cell proliferation, survival, movement and invasion in different malignancies [9–11]. In thyroid carcinoma, aberrant XB130 expression induces cell death and enhances apoptosis [12]. Interestingly, high XB130 protein level improves survival and sensitivity to 5-Fluorouracil in gastric cancer patients [13] while correlates with higher pathological grade and poorer prognosis of pancreatic ductal carcinoma [14]. Thus the association between XB130 and prostate cancer are worthy to be explored. Our previous findings demonstrated that low to moderate XB130 expression were seen in normal, hyperplastic and peritumoral prostate tissues, while elevated XB130 expression was displayed in prostate cancer tissues (Data not shown), but whether XB130 can affect cellular growth and invasion in prostate cancer is unclear. Thus in present study, we further investigated the linkage between XB130 and prognosis of prostate cancer and effects of XB130 on proliferation, invasion and metastasis of prostate cancer cells.

## RESULTS

### XB130 is a novel prognostic indicator for prostate cancer

**Figure 1:**
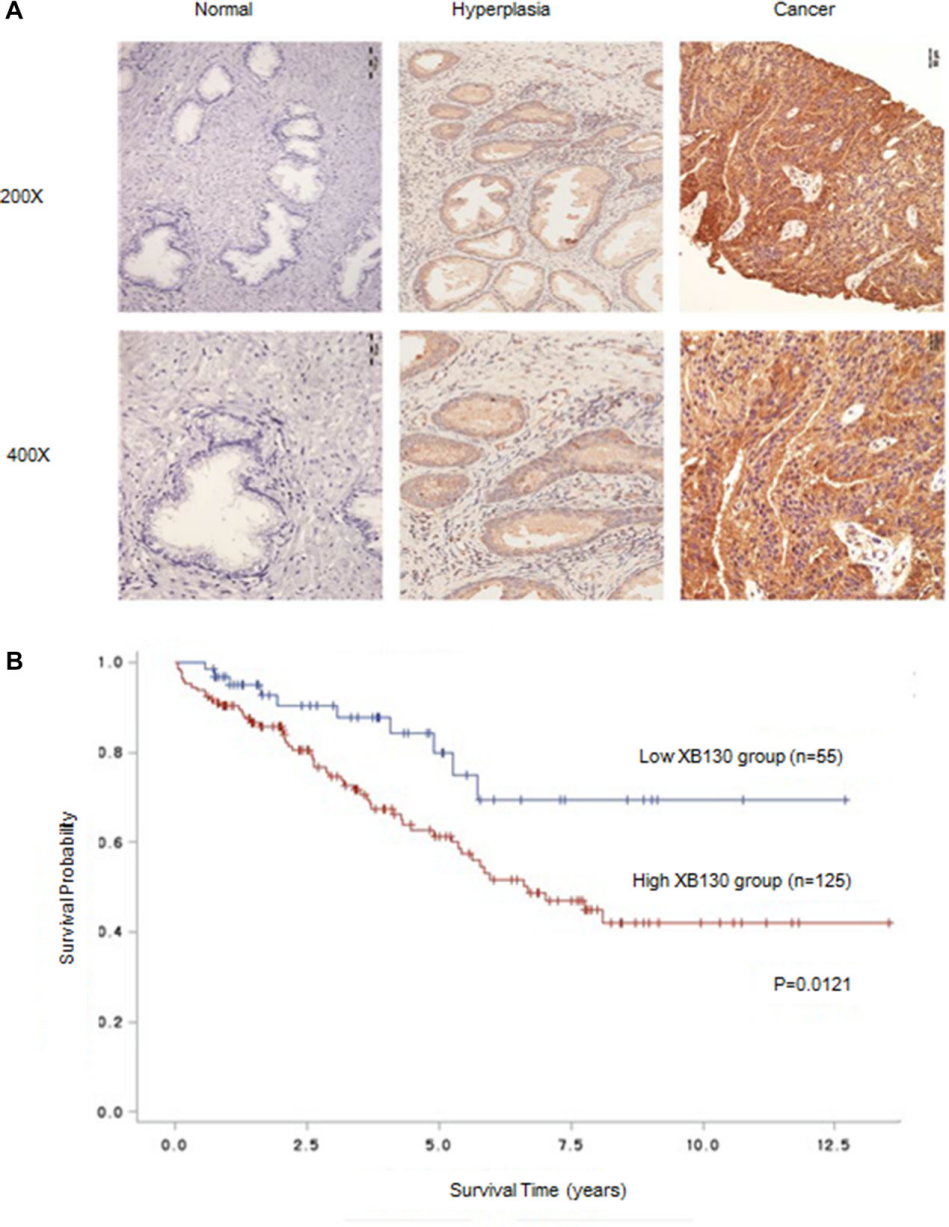
XB130 expression was correlated with prognosis of prostate cancer (**A**) Representative IHC-stained section of XB130 in different prostate tissues. XB130 expression was remarkably increased in prostate cancer tissues as compared with hyperplastic and normal tissues. (**B**) Kaplan–Meier analysis of the relation between XB130 and prognosis of prostate cancer.

We first examined XB130 expression in 210 cases of prostate cancer with pathological diagnosis. Consistent with our previous findings (data not shown), XB130 expression was remarkably increased in prostate cancer tissues as compared with hyperplastic and normal tissues (Figure 1A). As Table 1 indicated, XB130 was positively expressed in 85.6% specimens and significantly correlated with increased prostate specific antigen (PSA) (*p* = 0.006), free PSA (f-PSA) (*p* = 0.036), prostatic acid phosphatase (PAP) (*p* = 0.02) and T classification (*p* = 0.025). Kaplan–Meier analysis (Figure 1B) revealed that patients with highly expressed XB130 have significantly decreased survival rate than those with lower XB130 expression (*p* = 0.0121), which indicated that XB130 expression was inversely correlated with survival in prostate cancer.

**Table 1:** Relation between XB130 expression and clinicopathologic variables inprostate cancer patients

variables	low expression	high expression	total	t/χ^2^	*P*
gleason	6.24 ± 1.84	7.04 ± 1.51		–3.295	0.001
PSA	43.08 ± 68.68	79.39 ± 143.15			0.006
f-PSA	8.71 ± 13.70	15.20 ± 22.80			0.036
T classification					
1	11 (17.2%)	8 (5.5%)	19 (9.1%)	9.356	0.025
2	37 (57.8%)	102 (70.3%)	139 (66.5%)		.
3	8 (12.5%)	11 (7.6%)	19 (9.1%)		.
4	8 (12.5%)	24 (16.6%)	32 (15.3%)		.
PAP					
1	6 (9.7%)	4 (2.7%)	10 (4.8%)	7.866	0.02
2	16 (25.8%)	24 (16.4%)	40 (19.2%)		.
3	40 (64.5%)	118 (80.8%)	158 (76.0%)		.

### XB130 knockdown suppresses growth of prostate cancer *in vitro*

**Figure 2:**
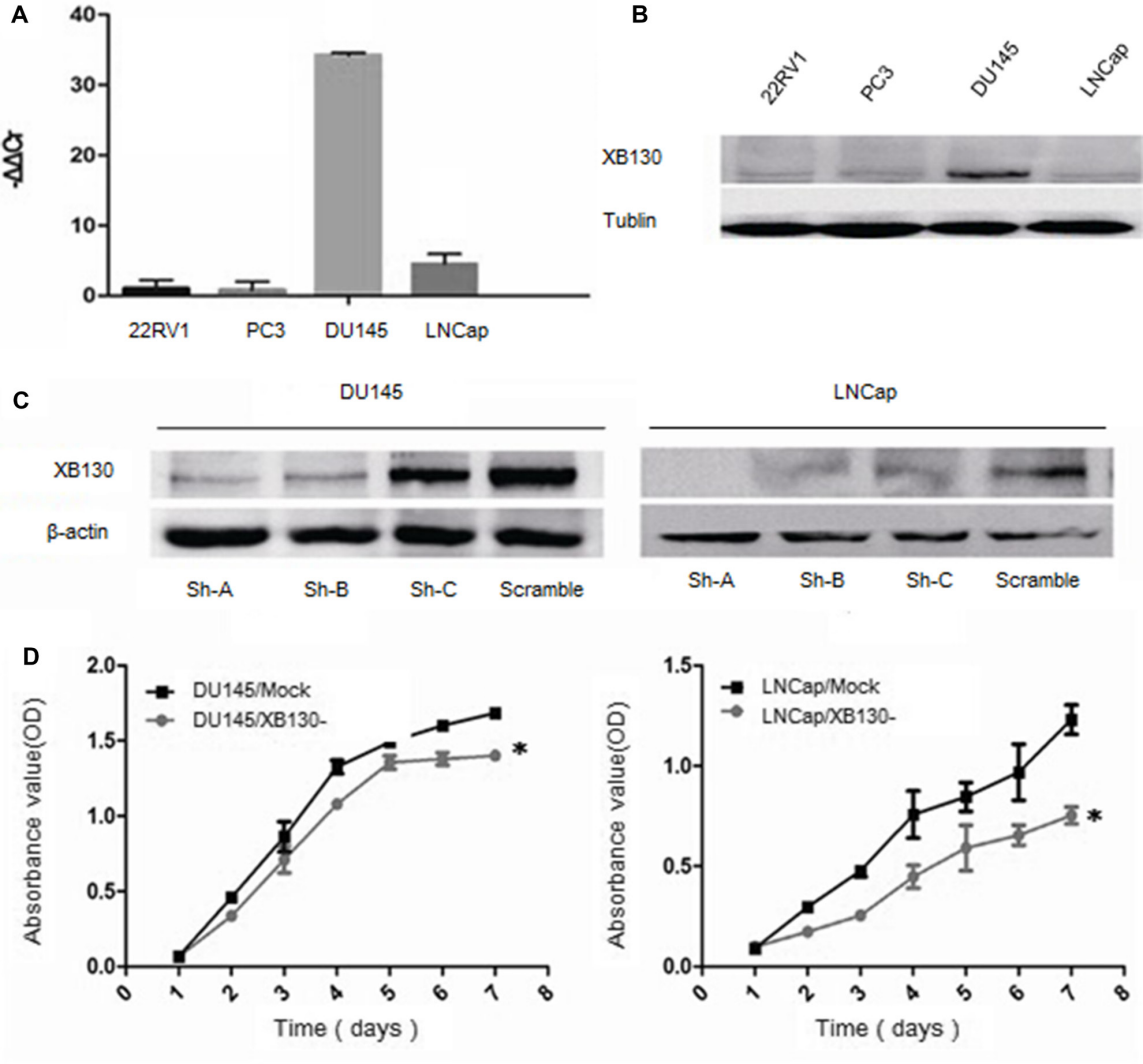
Knockdown of XB130 inhibited the proliferation of prostate cancer cell lines XB130 mRNA (**A**) and protein (**B**) expression in different prostate cancer cell lines. (**C**) Downregulation of XB130 with shRNA in DU145 and LNCap cells. (**D**) CCK8 assay indicated that XB130 knockdown inhibited cellular growth in DU145 and LNCap. Data are represented as mean +/− SEM. *P < 0.05 as compared to control groups.

XB130 mRNA (Figure 2A) and protein (Figure 2B) levels were detected in prostate cancer cell line 22RV1, LNCap, DU145, PC3, which showed that XB130 expressed in four prostate cancer cell lines at different levels. DU145 and LNCap expressed more XB130 in four prostate cancer cell lines than 22RV1 and PC3, thus were chosen for further knockdown studies.

**Figure 3:**
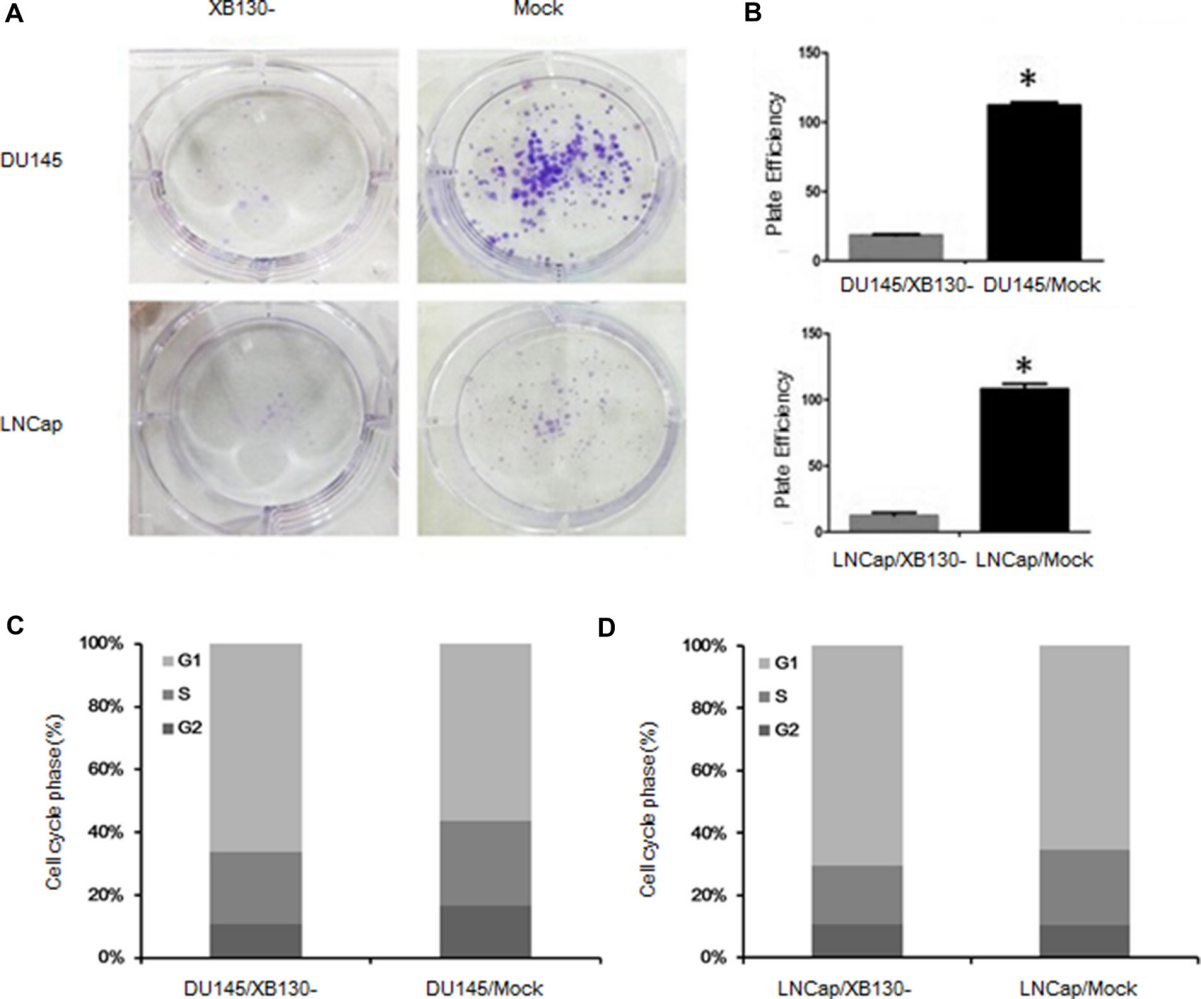
XB130 may restrain cellular proliferation through modulate transition of G1 to S phase in prostate cancer (**A**) XB130 knockdown decreased the colony formation of DU145 and LNCap. (**B**) Quantitative analysis of the colony form assay. Data are represented as mean +/− SEM. *P < 0.05 as compared to the control groups. (**C**) Flow cytometry analysis of cell cycle changes in DU145/XB130- and LNCap/XB130-. DU145/XB130- and LNCap/XB130- had significant shortened S phases than corresponding control groups. XB130 knockdown led to G1 phase arrest in both DU145 and LNCap.

To explore the effect of XB130 on proliferation of prostate cancer, we established two cell lines with downregulated XB130 expression and named as DU145/XB130- and LNCap/XB130-, while control cell lines were DU145/mock and LNCap/mock (Figure 2C). CCK8 assay (Figure 2D) showed that proliferation of DU145/XB130- was significantly slower than DU145/mock cells (F = 34.988, *p* = 0.02). Similar disparity was seen in LNCap/XB130-cells (Figure 2D, F = 43.644, *P* = 0.000). Colony formation assay (Figure 3A and 3B) indicated that, knockdown of XB130 in DU145 (F = 95.663, *p* = 0.000) and LNCap (F = 92.789, *p* = 0.000) could suppress the formation of colony of both groups comparing to DU145/mock and LNCap/mock. Downregulation of XB130 resulted in repression of colony formation by nearly 84% and 89% in DU145 and LNCap, respectively. Thus, decreased XB130 led to inhibitive proliferation of prostate cancer cell lines, which supported our previous conclusion that strong XB130 expression enhanced the growth of prostate cancer. In flow cytometry analysis (Figure 3C and 3D), DU145/XB130- (23.05% vs 26.92%, *p* = 0.04) and LNCap/XB130- (18.86% vs 24.12%, *p* = 0.03) displayed strikingly shorter S phase than control cells. Although not statistically significant, G1 phase prolongation were shown in DU145/XB130- (66.16% vs 56.43%, *p* = 0.2) and LNCap/XB130- cells (70.45% vs 65.43%, *p* = 0.3), which suggested that decreasing XB130 might subdue the transition of G1 to S phase in prostate cancer, which might be a cause for the restrain of cellular proliferation.

### XB130 knockdown inhibits tumor growth *in vivo*

**Figure 4:**
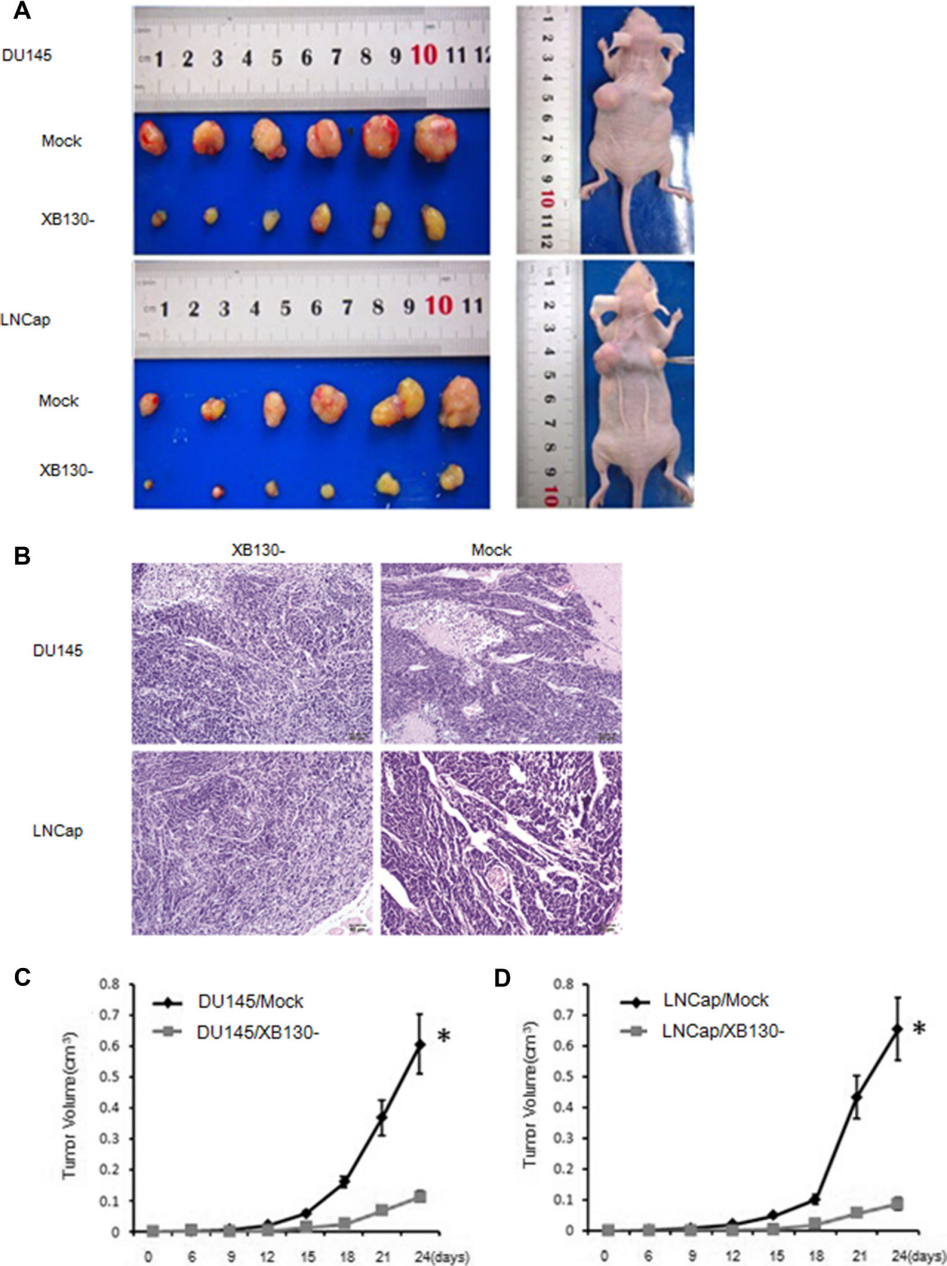
XB130 downregulation inhibited prostate cancer growth *in vivo* (**A**) DU145/XB130-and LNCap/XB130- tumors showed significant decreases in tumor volume comparing with control tumors on day 24 after injection. (**B**) Histology of DU145/XB130-and LNCap/XB130- tumors. Magnification, × 200 (**C**) Tumor growth curve of after injection of nude mice with DU145/XB130- or control cells. (**D**) Tumor growth curve of after injection of nude mice with LNCap/XB130- or control cells. Data are represented as mean +/− SEM. *P < 0.05 as compared to control groups.

We also investigated the effect of XB130 on the tumor growth using xenograft model in nude mice. Six days after subcutaneous injection of 10^7^ cells on the back of nude mice, we started to assess the tumor growth by measuring the tumor size when apparent tumors were seen on the back of mice. The tumor formation in the XB130 knockdown groups was significantly slower than in control groups. The tumors of knockdown groups were strikingly smaller when compared with control groups at day 18 and the difference became significant at day 21 after injection. As indicated in Figure 4A and 4B, remarkable suppression of tumor size and volume was seen in xenografts from DU145/XB130- (*p* = 0.03) and LNCap/XB130- (*p* = 0.02) when compared with control groups. In DU145/XB130- and LNCap/XB130- groups, the average tumor volumes reached 0.07 and 0.06 cm^3^ on day 24 after tumor cell inoculation, whereas control groups had average volumes of 0.37 and 0.43 cm^3^ (Figure 4C and 4D). These findings suggested that decreased XB130 restrained tumor growth *in vivo*.

### XB130 knockdown attenuates invasiveness of prostate cancer

**Figure 5:**
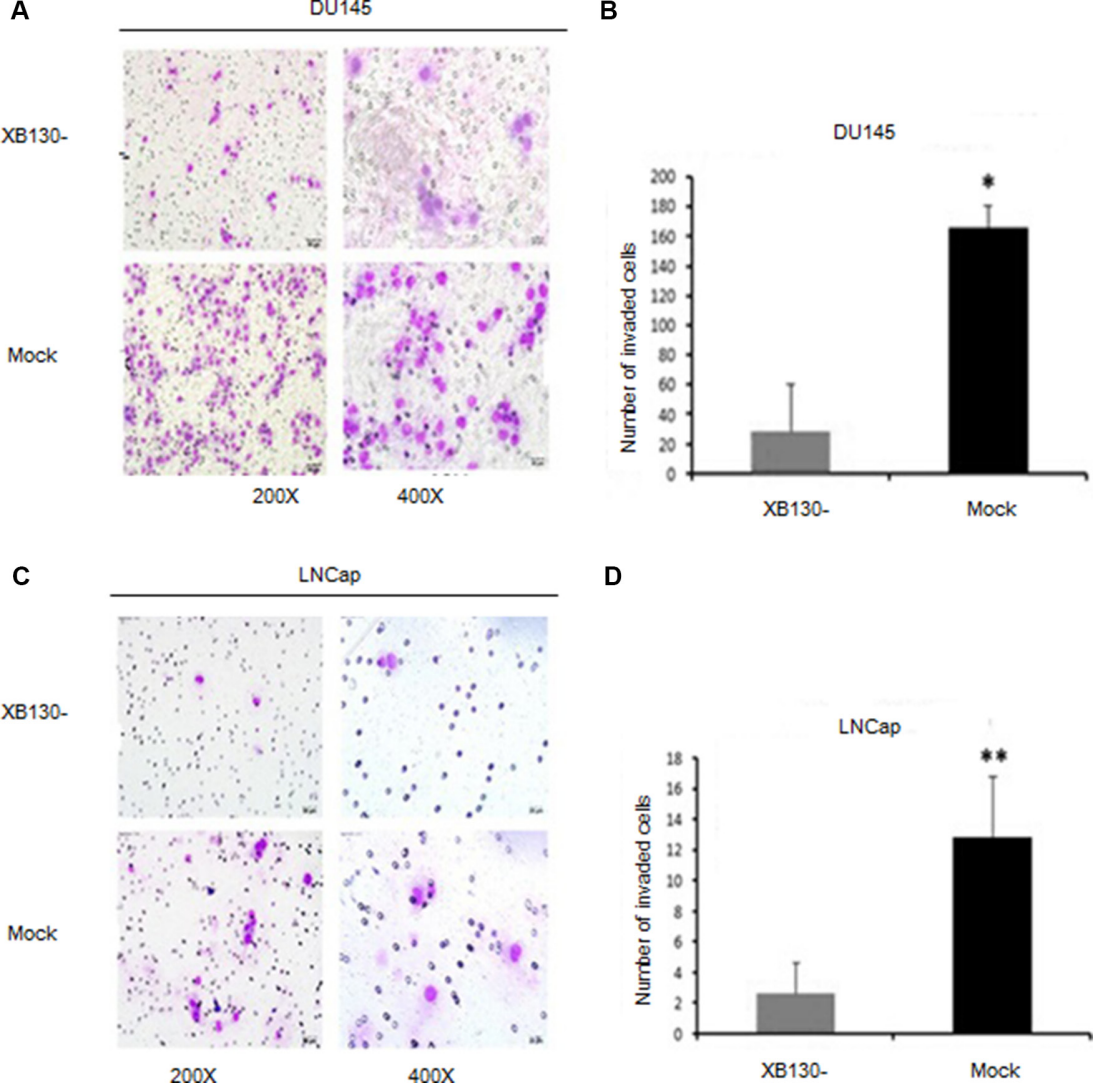
Reduced XB130 expression attenuated invasiveness of prostate cancer (**A**) XB130 downregulation inhibited invasiveness of DU145 in Matrigel. (**B**) Numbers of DU145/XB130- cells that pass through Matrigel was significantly less than control group. (**C**) XB130 downregulation suppressed invasiveness of LNCap in Matrigel. (**D**) Numbers of LNCap/XB130- cells that pass through Matrigel was significantly less than control group. Data are represented as mean +/− SEM.*P < 0.05 as compared to control groups. **P < 0.01 as compared to control groups.

To determine whether XB130 affects invasion of prostate cancer, we performed transwell assay with DU145 and LNCap cells. As shown in Figure 5A–5D, knockdown of XB130 (F = 83.922, *P* = 0.01) significantly inhibited invasion in DU145 cells when compared with control cells. Similar difference was also shown in LNCap cells (F = 92.164, *P* = 0.002). These data implied that endogenous XB130 also boosted invasion and metastasis of prostate cancer.

### XB130 stimulates Akt and EMT signaling

**Figure 6:**
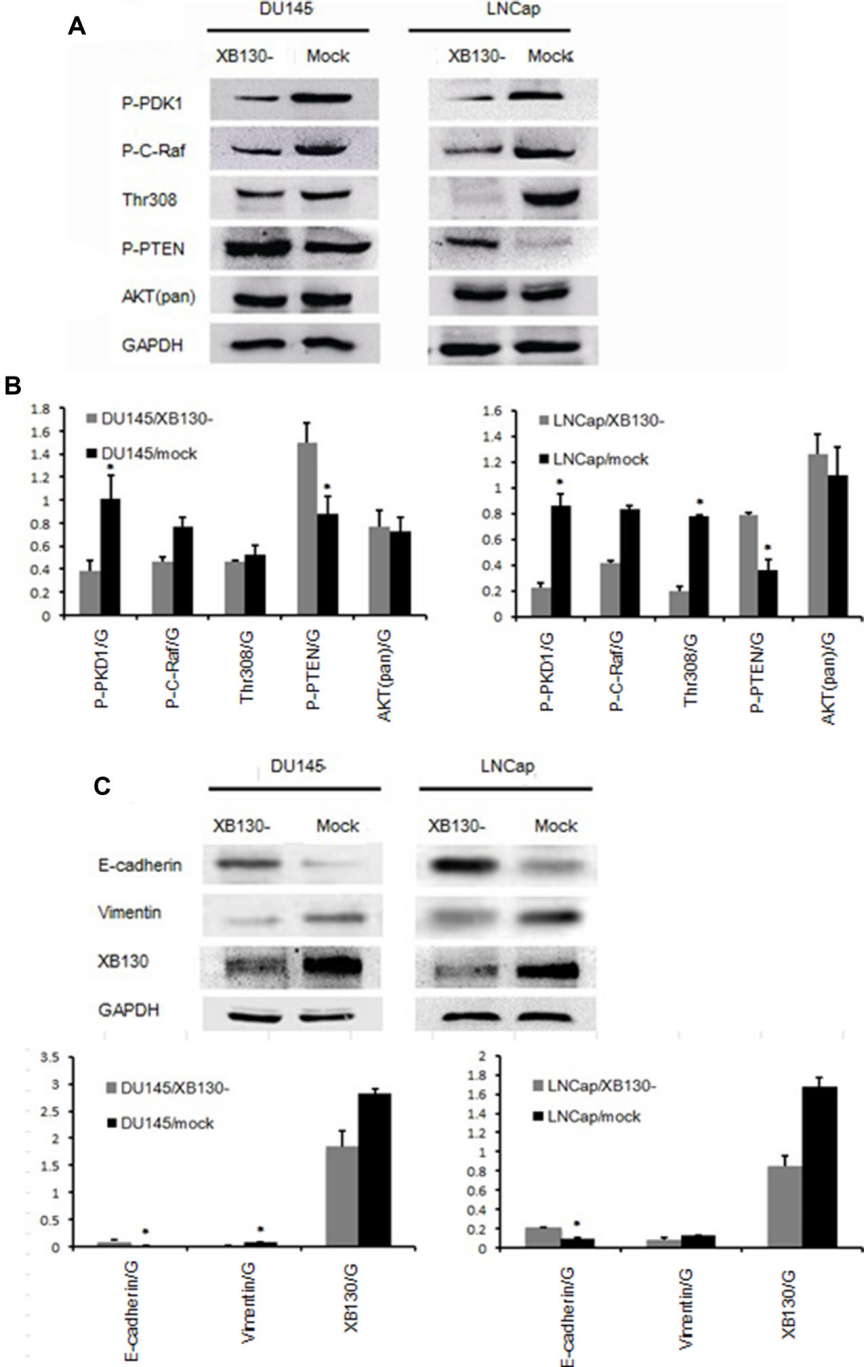
XB130 was involved in both Akt signaling and EMT process (**A**) Akt signaling altered with XB130 interference in both DU145 and LNCap. (**B**) Semi quantitative analysis of protein level changes in Akt pathway in DU145/XB130- and LNCap/XB130-. (**C**) EMT process was hindered by XB130 knockdown. (**D**) Semi quantitative analysis of protein level changes in EMT process in DU145/XB130- and LNCap/XB130-. Data are represented as mean +/− SEM. *P < 0.05 as compared to control groups.

In order to pursue the mechanisms of XB130 on the tumor progression, we detected potential downstream targets of XB130. Semiquantitative analysis of western blot (Figure 6A and 6B) showed that p-PDK1, p-C-Raf and Thr308 protein levels in DU145/XB130- and LNCap/XB130- significantly decreased, while p-PTEN was upregulated as compared to control cells. Interestingly, AKT (pan) expression didn't exhibit difference inXB130 knockdown cells. Two well-known markers of epithelial-mesenchymal transition (EMT) were also affected by XB130 abatement, with mesenchymal marker vimentin expression increased and epithelial marker E-cadherin decreased (Figure 6C and 6D).

## DISCUSSION

At present, only a few studies investigated the role of XB130 in cancer. In an immunohistochemistry analysis of 52 samples, 71.2% of the patients expressed high levels of XB130 in esophageal squamous cell carcinoma tissues, indicating that it may be a possible biomarker for diagnosis of esophagus cancer. It has also been proved to be an independent poor prognostic factor for survival of patients after operation [15]. Upregulation of XB130 and its significant connection with TNM stage and tumor differentiation were found in pancreatic cancer and breast cancer [16, 17]. But in gastric cancer, the expression profile seemed to be opposite. Low expression of XB130 was accompanied by shorter survival, disease-free period and diminished response to 5-fluorouracil therapy [13]. According to our results, XB130 level elevated in prostate cancer tissues when compared with adjacent tissues and 85.6% specimen shows positive staining, which suggests that XB130 could be a candidate marker assisting diagnosis of prostate cancer. Moreover, enhanced XB130 was correlated with a lower survival rate, which indicates it may serve as a poor prognostic factor in prostate cancer. The functions of XB130 in other cancers are worthy of more inspection.

Then we explored the possible effects and underlying mechanism of XB130 on prostate cancer cells. Several reports had demonstrated XB130 is involved in modulating proliferation and survival through regulation of essential cellular signaling [9–11]. Through RET/PTC pathway in thyroid cancer cells, XB130 diminution results in spontaneous apoptosis and enhancement of cell death with stimulant [11]. Downregulation of XB130 in thyroid cancer leads to remarkable alteration in gene expression profiling, including 57 genes involved in cell growth or survival and modulation of transcription. Pathway analysis suggests that the main molecular and cellular function of XB130 is modulation of proliferation, viability and cell cycle. Most common aliments associated with dysfunction of XB130 are all related to cancer [10]. Besides, inhibition of endogenous XB130 expression impairs the activity of c-Src, IL-8 signalizing and Akt phosphorylation in lung cancer cells [7]. Above findings indicates a vital role of XB130 in the growth, and survival of certain malignancies. Thus, we examined if XB130 also affect development of prostate cancer which could provide evidence for it being a candidate molecular target of novel therapy. CCK8 assay performed at different time points indicated that decreased XB130 remarkably attenuated viability of prostate cancer cells. Colony formation assay also supports this assumption by the result that XB130 knockdown caused a strikingly reduction in the number of colonies formed in DU145 and LNCap. Moreover, XB130 downregulation were accompanied by a diminished effect on S-phase cells and increased proportion of G1-phase cells, which is in line with previous findings in other solid tumors like lung cancer, thyroid cancer and esophagus cancer [7, 9, 10, 15]. Therefore, XB130 is an essential factor for proliferation of prostate cancer *in vitro* and G1 phase changes may contribute to this process. Further study on xenografts confirmed that XB130 knockdown inhibits growth of prostate cancer cells. Thus, XB130 overexpression may promote development of prostate cancer.

It has been reported that overexpression of XB130 boosts nicotine-derived nitrosamine ketone(NNK)-induced bronchial epithelial cell migration through enhancement of tyrosine phosphorylation and matrix metalloproteinase-14 translocation [18]. High-affinity lamellipodial F-actin reticular enables XB130 to take part in migration and invasion of thyroid cancer cells in a stimulus-induced fashion [7]. In this study, interference of XB130 significantly weakened the invasiveness of DU145 and LNCap, which suggested that XB130 expression may promote metastasis of prostate cancer.

Abnormal activation of PI3K/AKT pathway has been implicated in anti-apoptosis and dysplasia of many malignancies like colon caner, non-small cell lung carcinoma and lymphoma [19]. A YxxM motif in the N-terminal of XB130 facilitates the interaction with p85 subunit of PI3K, and therefore stimulates AKT activity in both thyroid and lung cancer cells [20]. Furthermore, downregulation of XB130 in thyroid cancer cells leads to decreased Akt phosphorylation at residue 473, which in turn inhibits cell cycle progression and cell growth [9]. In present analysis of PI3K/AKT pathway in DU145/XB130- and LNCap/XB130- cells, XB130 knockdown was accompanied by reduced expression of p-PDK1, thr308, p-C-Raf and upregulation of p-PTEN, which indicates that reducing XB130 deters Akt pathway in prostate cancer as in other cancers. Given the fact that AKT plays a key role in the cellular proliferation and survival and XB130 knockdown impedes AKT pathway, we concludes that XB130 downregulation may prohibit cell growth through inhibiting PI3K/AKT in prostate cancer.

PI3K/Akt signaling has been proved to be closely related with EMT events [21]. XB130 overexpression in squamous cancer cells SCC13 and SCC15 urge epithelial cells to gain characteristics of fibroblast, with decreased E-Cadherin and β-catenin and increased vimentin, which indicated occurrence of EMT event, reduction of cellular attachment and augmentation of mobility and invasiveness [22]. E-Cadherin was also reduced in DU145/XB130- and LNCap/XB130- associated with enhancement of vimentin expression, indicated that XB130 may promote migration and invasion of prostate cancer through mediating EMT by activating PI3K/Akt.

## MATERIALS AND METHODS

### Human specimens and cell lines

Two hundreds and ten paraffin-embedded prostate cancer samples and clinical information from April, 2000 to September, 2013 were obtained from consenting patients in General Hospital of Guangzhou Military Command of People's Liberation Army. The age range was 15 to 83 years. The experimental procedures were approved by the Research Ethics Committee of General Hospital of Guangzhou Military Command of People's Liberation Army.

Human prostate cancer cell line 22RV1, DU145, LNCap and human embryonic kidney cell line 293FT cells were purchased from Shanghai Cell Bank of Chinese Academy of sciences. 22RV1 and LNCap were cultured in RPMI-1640 with 10% fetal calf serum. DU145 and PC3 were cultured in F-12 medium with 10% fetal bovine serum. All cell lines were incubated in a 37°C incubator with 5% CO2.

### Immunohistochemistry (IHC)

IHC was performed with rabbit anti-XB130 polyclonal antibody (Abnova) and EnVisionTM plus kit (DAKO) according to manufacturer's instruction. Briefly, after deparaffinization and hydration, retrieve antigen with citrate buffer (pH 6.0) and quenched endogenous peroxidase activity with 0.3% hydrogen peroxide solution, the sections were incubated with primary antibody (anti-XB130, 1:100) for 2 hours at room temperature. After incubation with secondary antibody for 30 minutes at room temperature, the bound antibody was detected with DAB as substrate. As positive control, sections with vascular epithelium were immunostained. Sections stained with PBS instead of primary antibody were applied as negative control. We used an IHC scoring system from Engers et al. Two independent pathologists, both masked to the patients' clinical status, made these judgments.

### Knockdown of XB130 in DU145 and LNcap

DNA constructs for expression of V5- and GFP epitope tagged CycG2 fusion proteins in mammalian cells have been described (13, 15). Selection of shRNA target sites was done with RNAiDesignsoftware (Invitrogen). Oligonucleotide sequences are as described in Table 2.

**Table 2:** Oligonucleotide sequences of shRNAs targeting XB130

shXB130-A	Sense	GATCCCCGGAGCTAAAGGAAACCCTACTTTCAAGAGAAGTAGGGTTTCCTTTAG CTCCTTTTT
	Antisense	AGCTTAAAAAGGAGCTAAAGGAAACCCTACTTCTCTTGAAAGTAGGGTTTCCTT TAGCTCCGGG
shXB130-B	Sense	GATCCCCGCCGATAGGGTCTCCTGTATTTTCAAGAGAAATACAGGAGACCCTATC GGCTTTTT
	Antisense	AGCTTAAAAAGCCGATAGGGTCTCCTGTATTTCTCTTGAAAGTAGGGTTTCCTTTA GCTCCGGG
shXB130-C	Sense	GATCCCCGCTGAAGATCACACCGATGTTCAAGAGACATCGGTGTGATCTTCAGC TTTTT
	Antisense	AGCTTAAAAAGCTGAAGATCACACCGATGTCTCTTGAAAGTAGGGTTTCCTTTAG CTCCGGG
XB130-scramble	Sense	GATCCCCGCCAGCTTAGCACTGACTCTTCAAGAGAGAGTCAGTGCTAAGCTGG CTTTTT
	Antisense	AGCTTAAAAAGCCAGCTTAGCACTGACTCTCTCTTGAAAGTAGGGTTTCCTTTA GCTCCGGG

ShRNAs were inserted into linearized pGCSIL-GFP at AgeI and EcoRI sites. Lentiviruses were produced by transfecting 293FT cells of pGCSIL-GFP constructs containing shRNAs, pHelper1.0 and pHelper2.0 with Lipo2000 (Invitrogen). Supernatants were filtered and used for infecting DU145 and LNcap. Infection efficacy was observed by GFP expression under fluorescence microscope. Cell lines with over 80% efficacy were considered stable.

### RT-PCR

Total RNA was extracted with Trizol reagent (Invitrogen) and reversely transcribed according to the manufacturer's instruction (Invitrogen). Semi quantitative reverse transcription–polymerase chain reaction was used to measure XB130 mRNA levels using primers XB130-F (CTCCTCCGGCTTTACACCAAA) and XB130-R (GGCAAGCTGTTTCCGTTCTG), with GAPDH as control (GAPDH-F, TGTGGGCATCAATGGATTTGG; GAPDH-R, TGTGGGCATCAATGGATTTGG).

### Western blot

Cells were lysed in RIPA buffer and quantified with a BCA Protein Assay Reagent Kit (Pierce). 20 ug Cell lysates of total proteins were separated by SDS-PAGE, and transferred onto PVDF membranes (Roche). Blot then was probed with indicated antibodies and detected with an enhanced chemiluminescence detection kit (ECL) (Thermo). Band density was quantified using the ImageJ software (http://rsb.info.nih.gov/ij/). Rabbit polyclonal antibodies against XB130 (1:1000, PGT), pan-AKT (1:1000, Cell signaling), p-PDK1 (1:1000, Cell signaling), p-c-Raf (1:1000, Cell signaling), Thr308 (1:1000, Cell signaling), p-PTEN (1:1000, Cell signaling), p-GSK (1:1000, Cell signaling), E-cadherin (1:1000, Cell signaling), Vimentin (1:1000, Cell signaling) and mouse monoclonal antibodies against tubulin (1:4000, PGT) were used.

### Viability assay

For CCK8 assays, 5 × 10^3^ cells were plated in 96-well plates and observed for viability for 7 days. CCK8 method was used to detect the viability of cells according to the manufacturer's instructions. The absorbance at 570 nm was measured using a microplate reader. Five replicate wells were used for each group.

### Colony formation assay

For colony formation assay, 100 single cells from each group were seeded in 6-well plates and cultured at 37°C with 5% CO2 for 14 days before visualized by Giemsa stain. Colonies containing > 50 cells were scored, efficiency was calculated by the percentage of colony numbers in seeded cell numbers.

### Cell cycle analysis

Cells were fixed in 75% ethanol at –20°C for overnight. Washed pellets of fixed cells were resuspended in PBS containing 0.1% RNaseA (Fermentas) and 100 ul 10 ug/ml propidium iodide (Sigma) for 30 min at room temperature before flow cytometry using a FACScan (BD Biosciences) as described.

### Transwell assay

Cells were plated on the top matrigel-coated chamber of 24-well plate in medium without serum. Medium supplemented with 600 ul 10% FBS was used in the bottom chamber. After incubation at 37°C with 5% CO2 for 48 hours, cells on the lower membrane were fixed in 100% methanol and stained. Five random visual fields of each insert were counted under a microscope. Three independent experiments were conducted and the data were presented as the means ± SEM.

### *In vivo* xenograft animal model

BALB/c athymic nude mice were purchased from veterinary facilities of Southern Medical University. The mice were housed and maintained in accordance with the Animal Care and Use Guidelines of Southern Medical University under a protocol approved by the animal ethics committee of Southern Medical University. 10^7^ cells were trypsinized and washed into single cell suspension with serum-free medium. DU145/XB130- paired with DU145/mock and LNCap/XB130- paired with LNCap/mock were inoculated in nude mice respectively (XB130 knockdown cells on the right flank and control on the left counterpart). Mice were observed for 24 days before sacrifice by cervical dislocation. Tumors were measured every three days, then excised and fixed in formaldehyde. Six mice were in each group.

### Statistical methods

Correlation between XB130 expression and survival was analyzed with Kaplan-Meier method. RT-PCR, CCK8, cell cycle and transwell assay were compared with one-way ANOVA. *T*-test was used to compare Western blot, colony formation assay and tumor volumes. χ^2^ tests were conducted to assess correlations between XB130 expression and increase prostate specific antigen (PSA), free PSA (f-PSA), prostatic acid phosphatase (PAP) and T classification.
